# The Efficacy of Digital Interventions on Adherence to Oral Systemic Anticancer Therapy Among Patients With Cancer: Systematic Review and Meta-Analysis

**DOI:** 10.2196/64208

**Published:** 2025-04-16

**Authors:** Wan-Chuen Liao, Fiona Angus, Jane Conley, Li-Chia Chen

**Affiliations:** 1 Centre for Pharmacoepidemiology and Drug Safety, Division of Pharmacy and Optometry, School of Health Sciences, Faculty of Biology, Medicine and Health, The University of Manchester, Manchester Academic Health Science Centre, Oxford Road, Manchester, United Kingdom, 44 01613066000; 2 School of Dentistry, College of Medicine, National Taiwan University, Taipei, Taiwan; 3 Department of Pharmacy, The Christie NHS Foundation Trust, Manchester, United Kingdom

**Keywords:** efficacy, digital interventions, oral systemic anticancer therapy, medication adherence, cancer, oral, patients with cancer, therapy, systematic review, meta-analysis, care plans, medication, treatments, mobile app, mobile applications, mHealth, multimedia platforms, digital technology, self-reported, mobile phone

## Abstract

**Background:**

Digital interventions have been increasingly applied in multidisciplinary care plans to improve medication adherence to oral systemic anticancer therapy (SACT), the crucial lifesaving treatments for many cancers. However, there is still a lack of consensus on the efficacy of those digital interventions.

**Objectives:**

This systematic review and meta-analysis aimed to investigate the efficacy of digital interventions in improving adherence to oral SACTs in patients with cancer.

**Methods:**

This systematic review and meta-analysis followed the PRISMA (Preferred Reporting Items for Systematic Reviews and Meta-Analyses) statement guidelines. The protocol has been registered at PROSPERO (no. CRD42024550203). Fully published, randomized controlled trials (RCTs) in English on adults with cancer assessing digital interventions for improving adherence to oral SACTs were retrieved from MEDLINE, Embase, APA PsycINFO, and CINAHL Plus up to May 31, 2024. Adherence measures compared between digital intervention users and nonusers were extracted. The proportions of poor adherence were synthesized using a random-effects model. The pooled results were reported as the odds ratio and 95% CI. The heterogeneity was assessed with the *I*^2^ test (%). The mean difference and 95% CI were calculated from the mean adherence score and SD. A risk of bias assessment was conducted using version 2 of the Cochrane Risk of Bias Assessment Tool (RoB 2) for RCTs, which ensured that a quality assessment of all included studies was conducted as recommended by the Cochrane Collaboration.

**Results:**

This study included 13 RCTs on digital interventions for improving adherence to oral SACTs in patients with cancer. The 13 RCTs, published between 2016 and 2024, were conducted in the United States, South Korea, France, Egypt, Finland, Australia, Colombia, Singapore, and Turkey. The technologies used were mobile apps (n=4), reminder systems (n=4), telephone follow-ups (n=3), and interactive multimedia platforms (n=2). Adherence was measured by surveys (n=8), relative dose intensity (n=2), pill count (n=1), self-reported missed doses (n=1), a smart pill bottle (n=1), and urine aromatase inhibitor metabolite assays (n=1). Concerns regarding risk of bias primarily involved randomization, missing outcome data, and outcome measurement, including nonblinded randomization, subjective patient-reported data, and difficulties in distinguishing between missed appointments and actual medication nonadherence. Pooled results from 11 trials showed that digital technology users had significantly lower risk of poor adherence (odds ratio 0.60, 95% CI 0.47‐0.77). Two studies reported positive mean differences in adherence scores comparing digital intervention users and nonusers. However, due to considerable heterogeneity (*I*²=73.1%), it is difficult to make a definitive conclusion from the pooled results about the effect of digital interventions upon adherence to oral anticancer therapy.

**Conclusions:**

Digital intervention users exhibited significantly lower risk of poor oral SACTs adherence than nonusers. Acknowledging individual variation and tailoring digital technologies to prioritize patient needs is essential.

## Introduction

Medication adherence is a major public health concern, and nonadherence is responsible for 8% of global health expenditure and imposes a substantial economic burden on health care systems [1]. The advance in innovative treatments has led to an increasing number of cancers being classified as a long-term condition [2]. There is an increasing amount of research on measuring adherence [3], quantifying adherence rates in various drugs and cancer [4,5], investigating how to improve drug adherence [6], and identifying predictors of nonadherence [7].

Oral systemic anticancer therapy (SACT) has become increasingly accessible over the past 10 years, comprising 25% of oncology prescriptions globally [8] due to the advantages of being noninvasive, less intrusive, and more convenient [9]. However, they are prone to nonadherence as patients take medicines away from the medical setting. Many patients struggle to adhere to daily oral SACTs, with an adherence rate varying from 16% to 100% based on the settings and types of medicine [10].

Adherence is crucial to aiding successful patient outcomes of oral SACTs, while nonadherence can lead to disease progression, increased hospitalizations, and higher health care costs [11]. Factors such as complicated regimens, insufficient monitoring, poor communication, a lack of community support, mental health concerns, drug efficacy views, adverse effects, and financial load might contribute to nonadherence to oral SACT [6]. Clinicians may also neglect to mention the need for adherence and possible adverse effects, and patients may not have an adequate support system or understand the necessity of the medication [12]. Meanwhile, it has been asserted that interventions, including patient education and counseling, can improve treatment adherence [13].

Educational resources and various forms of communication have been used to build educational programs for patients in health care [14]. It is suggested that there is a link between continuous patient education and optimal adherence after a study showed that almost 50% of patients forgot their doctors’ instructions immediately after being told them [15]. Patient-centered care and individualized interventions incorporating digital strategies have emerged as promising directions for research and development [16].

Innovative digital approaches include telemedicine*,* which refers to the provision of clinical services remotely using communication tools such as video or telephone. It encompasses activities such as diagnosis, monitoring, advice, reminders, education, interventions, and remote admissions, offering benefits such as reduced travel costs and time [17]. Smart home technology is another app that integrates computing solutions into living spaces to provide various services, including health care. Using telecommunication and web technologies can involve remote monitoring systems that enable patients to receive support while remaining in their homes [18].

Recent evidence suggests that digital interventions improve medication adherence in patients with chronic conditions. A meta-analysis involving 11 studies across various diseases demonstrated that reminder-based interventions, including text messages, phone calls, and video calls, significantly improved adherence, with 65.94% of prescribed doses taken in the reminder groups compared with 54.71% in control groups (*P*=.04) [19].

In oncology, digital tools such as apps [20], text messages [21], mobile games [22], phone calls [23], and multimedia interactive information technologies [14] have been used to increase medical adherence. Specific benefits of the digital approach include aiding in treatment recall, promoting healthy lifestyle habits, and suggesting that patient-focused educational initiatives could enhance treatment adherence and quality of life [14,24]. According to Karaaslan-Eşer and Ayaz-Alkaya [25], digital apps are easy to use, safe, provide access to medical professionals, offer guidance on managing symptoms with real-time feedback, and send timely notifications to enhance treatment adherence.

However, previous publications on the digital approach to increasing adherence have been limited to targeted oral SACT [26], specific digital tools (such as mobile [27], app-based design [20], text message [28], or telemedicine [23]), and specific diseases [29,30], with previous reviews lacking synthesized results from a meta-analysis [31,32]. Furthermore, medications for cancer treatment differ from those for other chronic conditions, as dosing is often less stable. SACTs are often adjusted by clinicians in response to treatment-related side effects and disease progression, leading to fluctuating dosages that complicate patient adherence [33].

Given these unique challenges, further investigation is warranted to evaluate the efficacy of digital interventions on adherence, specifically for patients with cancer taking oral SACT. This knowledge gap can be explored by undertaking this systematic review and meta-analysis examining their efficacy.

## Methods

### Protocol Registration

This systematic review and meta-analysis followed the PRISMA (Preferred Reporting Items for Systematic Reviews and Meta-Analyses) statement guidelines (Multimedia Appendix 1) [34]. The protocol has been registered at PROSPERO (no. CRD42024550203). There were no deviations from the registered protocol.

### Selection Criteria

The inclusion and exclusion criteria of this study are summarized as follows (Table 1).

**Table 1.** Inclusion and exclusion criteria of this study.

	Inclusion criteria	Exclusion criteria
Population and conditions	Patients with cancer aged 18 years and older. Patients diagnosed with cancer. Patients with cancer taking oral SACTs^a^.	Patients with cancer including pediatrics, children, adolescents, neonates, or infants. Studies that include mixed age groups of participants with cancer. Patients with cancer taking nonoral SACTs^a^. Patients with cancer exclusively receiving injectable SACTs^a^.
Intervention and comparator	The use of digital interventions such as: Mobile apps Web-based platforms Wearable devices Telemedicine interventions Reminder systems (eg, text message reminders) Virtual support groups or web-based communities Comparator: standard or usual care without digital interventions.	Studies that use nondigital interventions to improve adherence. Studies with no suitable or appropriate comparator.
Outcome	Adherence measures such as: Medication possession ratio Proportion of days covered Self-reported adherence measures (eg, questionnaires and surveys) Pharmacy refill data Medication event monitoring systems (eg, smart pill bottles and electronic pill caps) Biological markers	The study does not contain outcome measures related to adherence. Adherence measures are based solely on subjective reporting (unless validated self-reported measures were used).
Study type	Human studies	Animal or in vitro studies
Language	English	Non-English language
Publication	Randomized controlled trials and clinical trials (comparative interventional trials)	Review papers, systematic reviews, meta-analyses, cross-sectional studies, case-control studies, pilot studies, feasibility studies, editorials, commentaries, letters, opinion pieces, conference abstracts, gray literature, and non–peer-reviewed sources.

a SACTs: systemic anticancer therapies.

### Types of Studies

Randomized controlled trials (RCTs) and clinical trials (nonrandomized, comparative interventional trials) were included. Review papers, systematic reviews, meta-analyses, cross-sectional studies, case-control studies, pilot studies, feasibility studies, editorials, commentaries, letters, opinion pieces, conference abstracts, gray literature, and non–peer-reviewed sources were excluded.

### Types of Participants

This study included participants who met the following criteria: (1) patients aged 18 years and older, (2) patients diagnosed with cancer, and (3) patients taking oral SACTs. Patients younger than 18 years, studies that included mixed-age groups of participants, patients with cancer taking nonoral SACTs, and patients with cancer exclusively receiving injectable SACTs were all excluded.

### Types of Interventions

The digital interventions were categorized according to the existing literature and the Cochrane Effective Practice and Organisation of Care (EPOC) taxonomy of health system interventions. EPOC outlined 4 categories of information and communication technology that health care organizations use for managing and delivering health care: health information systems, the application of information and communication technology, smart home technologies, and telemedicine [35].

To improve their adherence to oral SACTs, patients with cancer who used digital interventions, such as mobile apps, web-based platforms, wearable devices, telemedicine interventions, reminder systems (eg, text message reminders), virtual support groups, or web-based communities, were included. Studies using nondigital interventions to enhance adherence were excluded.

### Types of Outcome Measures

As there is no gold standard for measuring adherence and its associated outcomes, studies that reported adherence to oral SACTs, measured by various methods including self-reported adherence measures (such as the Morisky Medication Adherence Scale Score [36]), pharmacy refill data, medication event monitoring systems (including smart pill bottles and electronic pill caps), and biological markers, and presented as continuous or dichotomous data, such as the medication possession ratio [37], the proportion of days covered [37], or the proportion of adherence or nonadherence, were included in this review. Any studies that did not contain outcome measures related to adherence and studies that used adherence measures based solely on subjective reporting (unless validated self-reported measures were used) were excluded.

### Data Sources and Search Strategies

A comprehensive electronic database search was conducted on MEDLINE, Embase, APA PsycINFO, and CINAHL Plus from their inception to May 31, 2024, as this review began in June 2024. MEDLINE and Embase are widely recommended for studying health care interventions [38], while APA PsycINFO and CINAHL Plus, although narrower in scope, are also well suited for this field. These databases focus on subject-specific rather than population-based information. Although there is no established guideline for the number of databases to include in a search, the combination of 2 broad and 2 focused databases is considered appropriate for the subject area of this review. Various structured search strategies were used, using controlled vocabulary and keywords based on the study’s inclusion and exclusion criteria (Table 1) (Multimedia Appendix 2).

### Study Selection

The title and abstract of papers retrieved from the electronic databases search were first screened by 2 reviewers (FA and WCL) independently according to the selection criteria (Table 1) using the predesigned electronic screening form. Each paper was rated as “included,” “further check,” or “excluded.” The intraclass correlation coefficient (2-way mixed-effects model with absolute agreement [39]) and 95% CI were calculated for the consistency between 2 reviewers (FA and WCL) in record screening. Any discrepancy was resolved by discussing between reviewers and, if necessary, with a third reviewer (LCC) to reach a consensus. The full texts of potentially eligible papers were further reviewed independently by 2 reviewers (FA and WCL) to conclude the selection of studies.

### Data Extraction and Management

The data for each study were independently extracted by 2 reviewers (FA and WCL) using the standardized and piloted electronic data extraction sheet. Disagreements were adjudicated by a third reviewer (LCC). Study information (study title, lead author, country, and year of publication), study design, setting, targeted population (cancer and oral SACT), intervention (digital apps), comparison, outcome measures, and follow-up period were extracted. Study results, including continuous data (such as mean adherence scale score and SD) and dichotomous data (such as the proportion of adherent or nonadherent patients), were retrieved. If raw data are unavailable, risk ratio, hazard ratio, mean (SD), median (range) of adherence duration, or any other results that can be converted into raw data were extracted. Duplicates were identified using EndNote 20 (Clarivate Analytics) through its default 1-step auto-deduplication process, which applies the matching criteria of “author,” “year,” and “title.” This process was used to aid in screening the studies.

### Risk of Bias Assessment

Controlling the risk of bias in a systematic review is crucial, as bias can distort the true effect of interventions [40]. Quality assessment of all included studies was conducted using version 2 of the Cochrane Risk of Bias Assessment Tool (RoB 2) for RCTs as recommended by the Cochrane Collaboration [41]. By assessing bias across 5 critical methodological aspects of each RCT, namely, the randomization process, deviations from the intended intervention, missing outcome data, outcome measurement, and selection of reported results [41], the included studies were categorized into “’low risk of bias,” “some concerns,” or “high risk of bias” using the RoB 2 tool. The results were subsequently tabulated. Risk of bias assessment was conducted independently and in duplicate by the 2 reviewers (FA and WCL).

### Data Analysis

All outcomes were compared between the exposed group (digital intervention users) and the nonexposed group (those receiving standard care). The proportions of poor adherence were synthesized using a random-effects model (Der-Simonian and Laird method [42]). The pooled results were reported as odds ratio and 95% CI. The heterogeneity was assessed with the *I^2^* test (%). If appropriate, the mean difference and 95% CI of the adherence scale scores between the exposed and nonexposed groups were calculated and synthesized. The meta-analysis was conducted in STATA (Release 14; StataCorp LLC).

## Results

### Selection of Study

**Figure 1.**
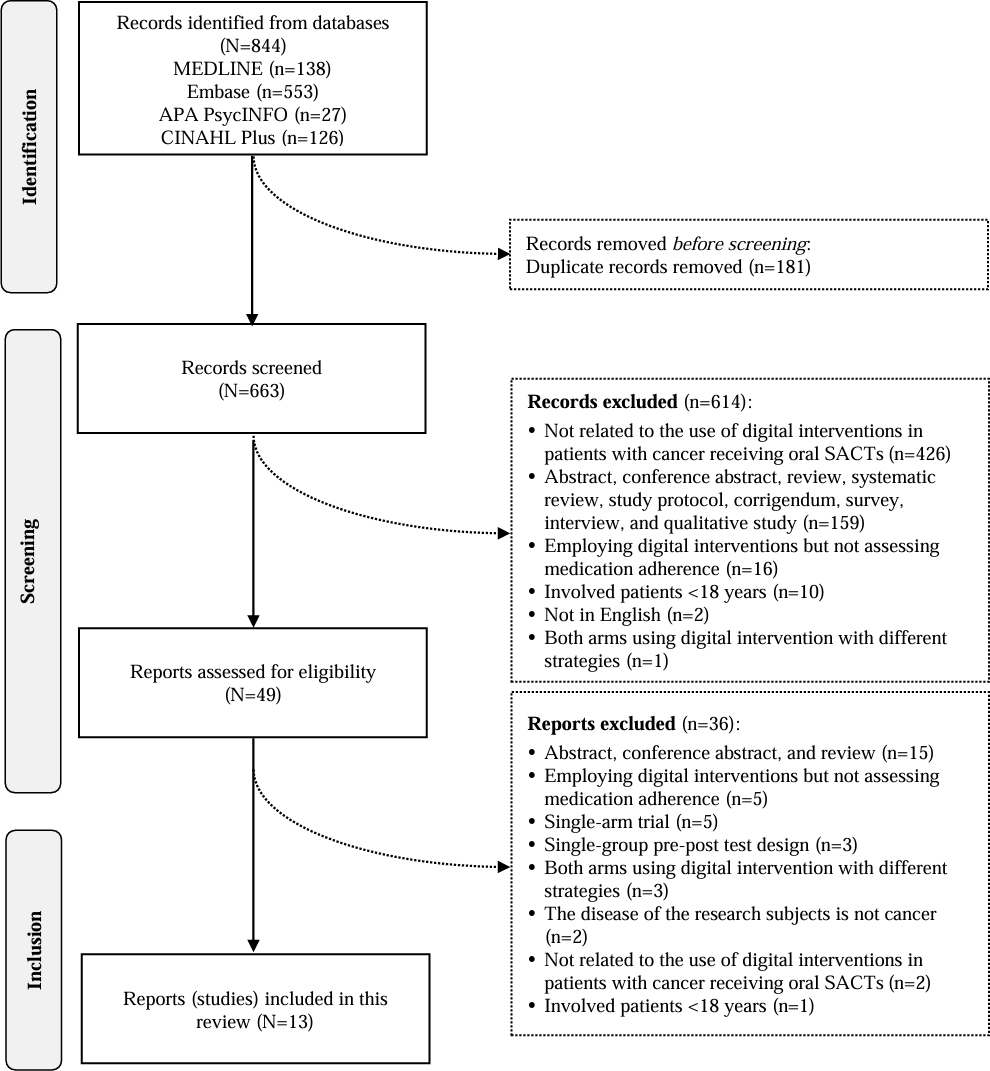
Selection of studies. APA: American Psychological Association; SACTs: systemic anticancer therapies.

Of the 844 records identified from the electronic databases search, 181 duplicates were deleted. After screening titles and abstracts, 614 records were removed due to the irrelevance to digital interventions in patients with cancer receiving oral SACTs (n=426), being not fully published original interventional papers (n=159), not assessing medication adherence (n=16), involving patients younger than 18 years (n=10), not being in English (n=2), and both arms using digital interventions (n=1). After the full-text screening of the remaining 49 studies, 36 were excluded, leaving 13 studies (2611 participants) for inclusion in this review (Figure 1). The intraclass correlation coefficient between the 2 reviewers (WCL and FA) is 0.886 (95% CI 0.868-0.902), indicating good consistency. Since both authors demonstrated consistency and agreement at the full-text screening stage, the intraclass correlation coefficient was calculated solely for the abstract screening.

### Characteristics of Study

The 13 included RCTs, published from 2016 to 2024, were conducted in various countries: the United States (n=3) [21,33,43], South Korea (n=2) [22,24], France (n=2) [44,45], Egypt (n=1) [23], Finland (n=1) [46], Australia (n=1) [47], Colombia (n=1) [14], Singapore (n=1) [48], and Turkey (n=1) [25]. The studies involved patients with breast cancer (n=5) [21,22,24,47,48], various types of cancer (n=5) [25,33,43-45], chronic myeloid leukemia (n=1) [46], colorectal or gastric cancer (n=1) [23], and multiple myeloma (n=1) [14]. Digital interventions included mobile apps (n=4) [22,24,25,43], reminder systems (n=4) [21,33,47,48], telephone follow-ups (n=3) [23,44,45] and interactive multimedia platforms (n=2) [14,46]. According to the EPOC taxonomy [35], 7 RCTs used smart-home technologies [22,24,25,33,43,47,48], 4 used telemedicine [23,33,44,45], and 2 used information and communication technology [14,46] (Table 2). There were 1305 patients in the digital intervention group and 1306 patients in the control group.

**Table 2.** Characteristics of included studies.

Author, year, country	Cancer type, age of patients(years)	Digital intervention		Control	Adherence measure
		Tools or technology and intensity of intervention	EPOC^a^		
[Bibr R47]Kekale et al (2016), Finland [46]	Chronic myeloid leukemia, median (range): 60 (25-83).	30-minute face-to-face counseling and multimedia interactive information technologies comprising a 5-minute video and daily text messages for 9 months.	Information and communication technology	Standard treatment	MMAS^b^
[Bibr R22]Kim et al (2018), South Korea [22]	Metastatic breast cancer, mean (SD): 50.9 (7.0)	Mobile game. Play the game for >30 minutes, 3 times weekly, for 3 weeks.	Smart-home technologies	Routine care	K-MARS^c^
Sikorskii et al (2018), United States [33]	Various types of cancer^d^, mean (SD): 61 (12).	Reminder phone calls consisting of daily adherence reminder calls.	Telemedicine	Standard care	RDI^e^
[Bibr R23]Eldeib et al (2019), Egypt [23]	Metastatic colorectal or gastric cancer, mean (SD): intervention group: 49.98 (10.7); control group: 44.8 (12.65)	Follow-up phone calls involving weekly phone calls for the 11 cycles of treatment.	Telemedicine	Standard care	Pill count method
[Bibr R44]Greer et al (2020), United States [43]	Various types of cancer^f^, mean (SD): 53.30 (12.91)	Mobile app with patients using the app for 12 weeks.	Smart-home technologies	Standard care	MMAS^b^
[Bibr R21]Hershman et al (2020), United States [21]	Early-stage breast cancer, median (range): 60.9 (30.7‐82.4)	Text message twice a week for 3 years.	Smart-home technologies	No text messaging	Urine test
[Bibr R49]Tan et al (2020), Singapore [48]	Breast cancer, median (range): 61 (32-80)	Text message weekly for 1 year.	Smart-home technologies	Standard care	SMAQ^g^
[Bibr R45]Bouleftour et al (2021), France [44]	Various types of cancer^h^, median (Q1-Q3): 70 (62-78)	Follow-up phone calls with calls at baseline, 3rd, 6th, 12th, and 24th weeks.	Telemedicine	Routine care	MMAS^b^
Karaaslan-Eser and Ayaz-Alkaya (2021), Turkey [25]	Various types of cancer^i^, mean (SD): intervention group: 60.33 (9.31); control group: 62.14 (9.97)	Mobile app, which was a weekly record of symptoms and severity for 6 months.	Smart-home technologies	Standard care	OCAS^j^
[Bibr R46]Mir et al (2022), France [45]	Various advanced or metastatic cancer^k^, median (range): 62 (20-92)	Follow-up by phone or internet (web portal) weekly for first month, biweekly from second to fourth month, and then 3 weekly from the fifth month onward.	Telemedicine	Usual care	RDI^e^ and questionnaire
[Bibr R24]Park et al (2022), South Korea [24]	Breast cancer, mean (SD): 53.33 (8.71)	Mobile app and smart pill bottle reminder with smart pill bottle reminder daily for 4 weeks.	Smart-home technologies	Usual care	Automatic smartphone records
[Bibr R48]Singleton et al (2023), Australia [47]	Breast cancer, mean (SD): 55.1 (11.1)	Text messages comprising 4 text messages weekly for 6 months.	Smart-home technologies	Usual care	Self-reported missed doses within the last 7 days
[Bibr R14]Guio et al (2024), Colombia [14]	Multiple myeloma, mean (SD): intervention group: 65.19 (10.45); control group: 62.25 (11.89)	Multimedia interactive information technologies. Contents are presented to patients and caregivers at the start of each 4-month cycle.	Information and communication technology	Conventional educational approach	MAQ^l^

a EPOC: Effective Practice and Organisation of Care.

b MMAS: Morisky Medical Adherence Scale.

c K-MARS: Korean version of the Medication Adherence Rating Scale.

d Breast, colorectal, gastrointestinal, leukemia, liver, lung, lymphoma, melanoma, myeloma, pancreatic, prostate, renal, sarcoma, brain, esophageal, and other cancer.

e RDI: relative dose intensity (defined as the ratio of the dose delivered over time to the prescribed dose intensity).

f Hematologic, non–small cell lung, breast, high-grade glioma, sarcoma, gastrointestinal, genitourinary, melanoma, and nongastrointestinal stromal tumor sarcoma.

g SMAQ: Simplified Medication Adherence Questionnaire.

h Hematologic, breast, prostate, pulmonary, kidney, colon, cerebral, rectum, sarcoma, and other cancers.

i Colorectal cancer, gastrointestinal stromal tumor, lung cancer, renal cell carcinoma, hepatocellular carcinoma, cholangiocarcinoma, breast cancer, pancreatic cancer, and glioblastoma.

j OCAS: Oral Chemotherapy Adherence Scale.

k Endocrine, breast, digestive, renal, central nervous system, sarcoma, gynecological, lung, hematological, melanoma, and other.

l MAQ: Medication Adherence Questionnaire.

### Quality Assessment

The 13 included RCTs raised concerns primarily related to the randomization process, missing outcome data, and outcome measurement; there were no high risks identified in any of the 5 areas of bias. The randomization was conducted by the principal investigator (KM) in one study [46] and lacked blinding in another [23]. In several studies, adherence outcomes were derived subjectively from patient-reported data via self-completed questionnaires [14,22,25,43-48]. In addition, challenges in differentiating missed appointments from actual medication nonadherence [21] and the possibility of smart pill bottles being opened without medication intake [24] further compounded measurement bias (Multimedia Appendix 3).

The challenges in recording outcome measures were found in 2 studies [21,24]. The authors of these RCTs made assumptions about the absence of urine samples as an indicator of nonadherence and the correlation between opening smart bottles and actual medication intake. While both studies used a sampling check or additional survey to support their assumptions, these diverse approaches contributed to increased heterogeneity and potential biases in this meta-analysis.

### Characteristics of the Interventions

Four studies used mobile apps to integrate educational materials into their platforms [22,24,25,43]. Although the app (ILOVEBREAST) by Kim et al [22] functioned as a game, it still served as an educational tool for patients. Standard features of these mobile apps include side effects and symptom management [22,25,43], lifestyle guidance [43], and addressing adherence concerns [24,43]. Two of these studies incorporated additional digital technologies into their mobile apps, such as smart pill bottle reminders [24] and integrated Fitbit for monitoring physical activity [43] (Multimedia Appendix 4).

Moreover, standard features across mobile apps and other digital technologies included disease management and patient education about specific cancer types. Three studies directly targeted adherence through their digital technologies, either by questioning patients about their adherence [23,44] or by measuring it [24]. The remaining studies indirectly addressed adherence by focusing on related features. Some text messages covered a variety of content related to not only medication adherence but also physical activity, healthy diet, well-being, side effects management, physician recommendations, and providing support [21,47]. In addition, 3 studies used digital interventions to identify problems, particularly symptoms and toxicities [25,44,45]. In 1 study, health care professionals were able to access patient data and communicate with nurse navigators via a web portal [45] (Multimedia Appendix 4).

The delivery mode of digital technologies in the 13 RCTs varied. Mobile apps involve self-administration by patients, constituting a passive delivery method, although 2 studies personalized the app experience with features such as customized medication dosing timetables and symptom recording [25,43]. Reminder systems, either via text message or phone call, were passively delivered through telecommunication companies [48] or an interactive voice response system [33], with reminders predominantly generic. Telephone follow-ups were tailored to individual patients and proactively delivered by trained nurses [44,45] or a single principal investigator [23]. Interactive multimedia platforms, although passively delivered, provided bespoke content. One study combined multimedia interactive platforms with face-to-face counseling sessions delivered by trained nurses [46] (Multimedia Appendix 4).

The duration of digital interventions in the 13 RCTs ranged from 3 weeks [22] to 3 years [21], with 1 study comprising 44 months in 11 undefined-length cycles [23]. Reminder systems were predominantly weekly, except for some studies conducted daily [33] or biweekly reminders [21]. Several studies used reminder systems to enhance adherence to oral SACTs. These systems varied, with some studies using smartphone messages [25,46,48], smart pill boxes [24], or telephone calls [33] to remind patients about their medication. Mobile apps were recommended for daily [22,24] or weekly use [25], except 1 study with unspecified frequency [33]. Telephone follow-ups varied from weekly [23] to less regular pattern [44,45]. One study combined follow-up phone calls with a web portal for web-based communication and patient information sharing [45]. Multimedia interactive platform engagement varied from monthly [14] to unspecified frequencies [46], with text messages being sent daily in 1 study [46] (Table 2).

### Adherence Measurement

Adherence was the primary outcome in 11 RCTs, while 2 studies assessed it as a secondary outcome [45,47]. Various subjective measures, including surveys [14,22,25,43-46,48], relative dose intensity (RDI) [33,45], pill count [23], self-reported missed doses [47], and a smart pill bottle [24], were used across the 13 RCTs. One study used a more objective measure of adherence using time-to-adherence failure, defined by urine aromatase inhibitor metabolite assay results [21] (Table 2).

### Adherence Rate

The pooled result from 11 studies [14,21,23-25,33,43,45-48] showed that users of digital technology had a significantly lower risk of poor adherence to oral SACTs than nonusers (odds ratio 0.60, 95% CI 0.47-0.77; *I*^2^=73.1%) (Table 3). A trend was observed where smaller studies favored the digital intervention group [14,25,46], while larger studies favored the control group or showed no significant difference [21,33,43,45,48]. However, definitive conclusions cannot be drawn due to substantial heterogeneity (*I*²=73.1%) [40]. In 1 study, only the proportion of medium adherence was reported, with no significant difference observed between the intervention (92/183, 77.2%) and control (91/183, 81.3%) groups [44].

**Table 3.** Proportion of patients with poor adherence in the included studies.

Study	Type of digital technology	Follow-up	Event rate^a^	Odds ratio (95% CI)
Kekäle et al (2016) [46]	Face-to-face counseling Interactive multimedia platforms	9 months	1/35 vs 9/33	0.08 (0.01‐0.66)
Sikorskii et al (2018) [33]	Reminder phone calls Disease self-management tool kits	12 weeks	0/106 vs 0/108	1.02 (0.02‐51.82)
Eldeib et al (2019) [23]	Follow-up phone calls	11 cycles	0/44 vs 3/38	0.13 (0.01‐2.73)
Greer et al (2020) [43]	Mobile app	12 weeks	11/80 vs 20/86	0.53 (0.23‐1.18)
Hershman et al (2020) [21]	Text message	3 years	238/290 vs 268/313	0.77 (0.50‐1.19)
Tan et al (2020) [48]	Text message	1 year	59/123 vs 55/121	1.11 (0.67‐1.83)
Karaaslan-Eser and Ayaz-Alkaya (2021) [25]	Text message	6 months	16/38 vs 28/39	0.29 (0.11‐0.74)
Mir et al (2022) [45	Follow-up by phone or internet (web portal)	6 months	15/255 vs 26/265	0.57 (0.30‐1.11)
Park et al (2022) [24]	Mobile app integrated with a smart pill bottle reminder	4 weeks	1/30 vs 3/27	0.28 (0.03‐2.83)
Singleton et al (2023) [47]	Text message	6 months	3/42 vs 8/47	0.38 (0.09‐1.52)
Guio et al (2024) [14]	Interactive multimedia platforms	At least 100 days following transplantation or 3 months after maintenance	1/16 vs 13/16	0.02 (0.01‐0.17)
Overall	N/A^b^	N/A	345/1059 vs 433/1093	0.60 (0.47‐0.77); *I*^2^=73.1%

a Event rate refers to the proportion of poor adherence in each study, measured by the specific method used in the study. Digital intervention users versus nonusers. Some event rate values have been converged based on the adherence data provided by studies.

b N/A: not applicable

### Adherence Scale Score and RDI

Two studies reported adherence scale scores [22,44]. Although the results were not pooled, the mean difference was calculated (Table 4). These 2 studies generated positive mean differences, indicating that digital technology users experienced an increase or improvement in oral SACT adherence compared with nonusers. The mean (SD) of the RDI for the intervention group and the control group were 0.89 (0.03) (n=122) and 0.92 (0.03) (n=117) in one study [33], and 0.84 (0.26) (n=255) and 0.80 (0.21) (n=265) in another study [45]. A value of RDI<0.8 indicated underadherence, as reported in 1 study [33].

**Table 4.** Adherence scale score and mean difference of the included studies.

Study	Digital technology	Follow-up	Adherence scale	Mean (SD) score^a^	Mean difference^b^ (95% CI)
Kim et al (2018) [22]	Mobile game	3 weeks	Korean version of the medication adherence rating scale	7.6 (0.7) (n=34) vs 6.5 (0.5) (n=38)	1.10 (0.82-1.38)^c^
Karaaslan-Eser and Ayaz-Alkaya (2021) [25]	Text message	6 months	Oral chemotherapy adherence scale	81.22 (8.05) (n=38) vs 73.36 (10.44) (n=39)	7.86 (3.81-11.91)^c^

a Digital intervention users versus nonusers.

b Mean difference represents the adherence score difference between digital intervention users and standard care patients, with higher scores indicating better adherence.

c *P*<.01.

## Discussion

### Principal Results

This study investigated the efficacy of digital interventions in improving adherence to oral SACTs and found that digital intervention users had a significantly lower risk of poor adherence to oral SACTs than nonusers. In addition, digital technology users demonstrated improved or increased adherence scores compared with nonusers.

Interactive and patient-focused digital supports have revolutionized the possibilities for improving medication adherence [16]. An overview of reviews indicates that incorporating digital technologies with direct clinician contact is likely to increase adherence [31]. A systematic review confirmed the efficacy of digital interventions in improving short-term treatment adherence among patients with cancer receiving oral chemotherapy [32]. Our pooled meta-analysis results also support this, as they showed a significantly reduced risk of poor adherence to oral SACTs among users of digital tools.

The efficacy of digital tools in achieving success can be attributed to various factors, for example, providing instructional resources, dosage aids, engagement with health care providers, digital medicine, self-monitoring, and quickly implementable technical methods [16]. Patient awareness of their drug regimen and the goals, benefits, and potential adverse events is critical for optimal adherence [49]. Digital can offer medication information and instructional help as educational resources [22,24,25,43]. Digital-based interventions such as personalized dosing schedules help patients organize and improve drug adherence [43]. Face-to-face counseling, proposed as a single consultation experience, was also included in our review for its potential to enhance patient adherence [46,50].

### Implications

Medication adherence is crucial in oncology therapy, yet low adherence rates, as low as 14% for some cancer regimens, significantly impact patient health outcomes and strain health care systems and budgets [51]. This indicates that personalized interventions may improve adherence [51,52]. With more than 4.57 billion web users globally, 91% are accessing it via mobile devices, and smartphone usage—projected to increase by 8% annually [53], as well as digital health tools including phones and wearable devices—offer promising avenues for enhancing health care outcomes, cost-effectiveness, and patient acceptance [27].

Telemedicine offers greater flexibility than in-person interventions, allowing for addressing nonadherence wherever and whenever it occurs, such as between appointments or outside of clinic settings [54]. Telemedicine for reminder and follow-up phone calls was also a method of implementation used in several studies examined [23,33,44]. Digital medicine involves tools such as electronic pill bottles and wearable electronic devices. These devices enhance adherence and can track when containers are opened, although this does not verify intake [55]. Moreover, digital treatments may have drawbacks, including the cost and time needed for transferring or connecting with electronic equipment [16].

One study investigated whether using 1 or 2 digital tools improved adherence [56]. Both groups received weekly automated voice responses over 8 weeks, with the intervention group receiving additional daily text messages for 21‐28 days. Results suggested that the extra text messages improved adherence and symptom management in patients taking oral anticancer agents. Another similar study showed that additional text messages could positively impact patients by promoting behavior change and improving self-care [28]. This highlights the potential for diverse clinical outcomes with varying types and quantities of digital tools.

Furthermore, social inequality is often correlated with the reduced use of digital technology in health care, contributing to a digital health divide [57]. For instance, older adults are less likely to use the web [58] or smartphones [59], and individuals with lower incomes face greater barriers to web access [60]. This inequality results in disparities in access to digital tools and hampers the implementation of digital interventions in health care [61]. To enhance accessibility, patients and health care professionals need to be involved in the development of these interventions, ensuring that they meet the needs of diverse patient populations. In addition, educational campaigns should aim to raise awareness and provide training on digital tools while also challenging stereotypes about older adults’ technological capabilities and reinforcing patients’ confidence in maintaining their privacy when using such interventions [61].

### Strengths and Limitations

This review focuses on managing medication adherence at home for patients with cancer who are prescribed oral SACTs. All studies included are RCTs, considered the gold standard for measuring intervention efficacy [62]. We excluded single-group pre-post test designs to ensure randomization and aimed to cover various contemporary digital tools to assess their efficacy on medication adherence. One study had a 3-year follow-up, offering valuable insights into long-term impact [21]. The pooled meta-analysis results provide an integrated understanding of digital tools’ efficacy in supporting medication adherence among patients with cancer.

While digital interventions hold promise, we acknowledge several limitations in this study, including various cancer types and oral SACT classes introducing disease uniqueness and drug response variability, potentially impacting medication adherence and intervention efficacy.

Despite including only RCTs, these studies exhibited considerable variability in research design, data collection methods, outcome measures, and the digital interventions used, as well as diversity in the cancer types investigated. The inability to conduct a patient-blinded experiment due to patient expectations of additional digital support is recognized [23,25]. Follow-up phone calls by different health care professionals may introduce bias [44,45]. Furthermore, reliance on subjective self-monitoring or self-reporting for medication adherence evaluation poses potential errors [24,33,46]. Small sample sizes in some trials may limit statistical power and significance between intervention and control groups.

This heterogeneity is inherent to the subject matter [63]. Methodological heterogeneity was notable (*I*²=73.1%), but it was accounted for by using a random-effects model in the meta-analysis, which assumes a normal distribution of underlying effects [40]. Also, due to the significant heterogeneity, the publication bias assessment test was not conducted to avoid presenting potentially misleading results. Acknowledging these limitations is crucial for interpreting the research results and allows readers to evaluate the significance and scope of the study more comprehensively. Another limitation of the study was that subgroup analyses were not conducted due to lack of data. This could have been used to investigate heterogeneous results or ask specific questions about a cancer type or intervention type.

This review included a variety of adherence and outcome measures due to the lack of consensus on these metrics. While self-reported adherence may be less robust due to recall bias and social desirability effects [64], only those studies using validated tools widely accepted in adherence research were included. Although these tools facilitate low-burden data collection, self-reported adherence may not always accurately reflect actual behavior, necessitating cautious interpretation of results. This diversity in outcome measures provides a comprehensive view of adherence-related consequences, which is crucial for understanding the broader context of digital interventions but may also complicate the ability to draw definitive conclusions.

Cancer populations encompass low-, middle-, and high-income regions globally, each with varying access to digital technologies and health care systems. Most studies have been conducted in high-income regions, which limits the generalizability of the results to low- and middle-income areas. In addition, the limited and diverse regional patient inclusion across these studies may further restrict the applicability of the findings to broader conditions [23-25,43].

### Recommendations

Future interventions should be developed that focus on patient-centered, motivation-driven, and culturally adapted digital tools and be tailored for individuals with different types of cancer or oral SACTs. Efforts should focus on minimizing the threshold and difficulties associated with using digital tools and ensuring accessibility and ease of implementation for patients of all ages. Investigating patients’ preferences for digital interventions could also increase usage rates. Monitoring health care professionals’ responses and perspectives on digital interventions, alongside tracking patients’ medication adherence, would provide valuable insights. To prevent alert fatigue [21], future research could explore optimal timing and frequency for implementing digital interventions. Qualitative studies could be conducted to delve deeper into the experiences of digital intervention users in real-world therapeutic settings, complementing quantitative findings.

### Conclusions

Considering the growing use of oral SACTs and their higher patient acceptance over intravenous therapy, addressing medication adherence is vital in clinical oncology. Digital interventions offer effective support, enhancing adherence to oral SACTs and improving treatment outcomes while providing convenience for patients. This study highlights the significant benefits of digital technology in promoting adherence. Future research should focus on refining and personalizing digital tools to better meet individual patients’ needs.

## Supplementary material

10.2196/64208 Multimedia Appendix 1 PRISMA 2020 checklist.

10.2196/64208 Multimedia Appendix 2 Electronic search strategy.

10.2196/64208 Multimedia Appendix 3 The risk-of-bias assessment for the randomized controlled trials.

10.2196/64208 Multimedia Appendix 4 Characteristics of interventions.

## Abbreviations

EPOC: Effective Practice and Organisation of Care
PRISMA: Preferred Reporting Items for Systematic Reviews and Meta-Analyses
RCT: randomized controlled trial
RDI: relative dose intensity
RoB 2: Risk of Bias Assessment Tool
SACT: systemic anticancer therapy
