# Supplementary material for: The Efficacy of Digital Interventions on Adherence to Oral Systemic Anticancer Therapy Among Patients With Cancer: Systematic Review and Meta-Analysis
Source: JMIR Cancer. 2025 Apr 16;11:e64208. doi: 10.2196/64208 (PMC12017607; doi:10.2196/64208)
Supplement: Multimedia Appendix 2 [file cancer-v11-e64208-s002.docx]

Appendix 2. Electronic search strategy

**MEDLINE**

1 Neoplasms/

2 oncolog*.mp.

3 neoplas*.mp.

4 cancer.mp.

5 cancer*.mp.

6 tumo*.mp.

7 tumou*.mp.

8 carcino*.mp.

9 malignan*.mp.

10 1 or 2 or 3 or 4 or 5 or 6 or 7 or 8 or 9

11 Antineoplastic Agents/

12 Anti-cancer therapies.mp.

13 medication*.mp.

14 oral drug.mp.

15 capsule*.mp.

16 Tablets/

17 pill*.mp.

18 11 or 12 or 13 or 14 or 15 or 16 or 17

19 Medication Adherence/

20 Adherence.mp.

21 Patient Compliance/

22 Treatment Refusal/

23 Self Administration/

24 non-adherence.mp.

25 noncompliance.mp.

26 non-compliance.mp.

27 non-adherence.mp.

28 persistence.mp.

29 concordance.mp.

30 19 or 20 or 21 or 22 or 23 or 24 or 25 or 26 or 27 or 28 or 29

31 Telemedicine/

32 Digital.mp.

33 digital interventions.mp.

34 telemedic*.mp.

35 eHealth.mp.

36 mHealth.mp.

37 Digital Health/

38 Internet-Based Intervention/

39 online therapy.mp.

40 Digital therapy.mp.

41 Mobile Applications/

42 apps.mp.

43 smart pill dispensers.mp.

44 Text Messaging/

45 Web-based platforms.mp.

46 Wearable Electronic Devices/

47 Virtual support groups.mp.

48 online communities.mp.

49 31 or 32 or 33 or 34 or 35 or 36 or 37 or 38 or 39 or 40 or 41 or 42 or 43 or 44 or 45 or 46 or 47 or 48

50 10 and 18 and 30 and 49

**EMBASE**

1 Neoplasms/

2 oncolog*.mp.

3 neoplas*.mp.

4 cancer.mp.

5 cancer*.mp.

6 tumo*.mp.

7 tumou*.mp.

8 carcino*.mp.

9 malignan*.mp.

10 1 or 2 or 3 or 4 or 5 or 6 or 7 or 8 or 9

11 Antineoplastic Agents/

12 Anti-cancer therapies.mp.

13 medication*.mp.

14 oral drug.mp.

15 capsule*.mp.

16 Tablets/

17 pill*.mp.

18 11 or 12 or 13 or 14 or 15 or 16 or 17

19 Medication Adherence/

20 Adherence.mp.

21 Patient Compliance/

22 Treatment Refusal/

23 Self Administration/

24 non-adherence.mp.

25 noncompliance.mp.

26 non-compliance.mp.

27 non-adherence.mp.

28 persistence.mp.

29 concordance.mp.

30 19 or 20 or 21 or 22 or 23 or 24 or 25 or 26 or 27 or 28 or 29

31 Telemedicine/

32 Digital.mp.

33 digital interventions.mp.

34 telemedic*.mp.

35 eHealth.mp.

36 mHealth.mp.

37 Digital Health/

38 Internet-Based Intervention/

39 online therapy.mp.

40 Digital therapy.mp.

41 Mobile Applications/

42 apps.mp.

43 smart pill dispensers.mp.

44 Text Messaging/

45 Web-based platforms.mp.

46 Wearable Electronic Devices/

47 Virtual support groups.mp.

48 online communities.mp.

49 31 or 32 or 33 or 34 or 35 or 36 or 37 or 38 or 39 or 40 or 41 or 42 or 43 or 44 or 45 or 46 or 47 or 48

50 10 and 18 and 30 and 49

**APA PsycInfo**

1 Neoplasms/

2 oncolog*.mp.

3 neoplas*.mp.

4 cancer.mp.

5 cancer*.mp.

6 tumo*.mp.

7 tumou*.mp.

8 carcino*.mp.

9 malignan*.mp.

10 1 or 2 or 3 or 4 or 5 or 6 or 7 or 8 or 9

11 Antineoplastic Agents/

12 Anti-cancer therapies.mp.

13 medication*.mp.

14 oral drug.mp.

15 capsule*.mp.

16 Tablets/

17 pill*.mp.

18 11 or 12 or 13 or 14 or 15 or 16 or 17

19 Medication Adherence/

20 Adherence.mp.

21 Patient Compliance/

22 Treatment Refusal/

23 Self Administration/

24 non-adherence.mp.

25 noncompliance.mp.

26 non-compliance.mp.

27 non-adherence.mp.

28 persistence.mp.

29 concordance.mp.

30 19 or 20 or 21 or 22 or 23 or 24 or 25 or 26 or 27 or 28 or 29

31 Telemedicine/

32 Digital.mp.

33 digital interventions.mp.

34 telemedic*.mp.

35 eHealth.mp.

36 mHealth.mp.

37 Digital Health/

38 Internet-Based Intervention/

39 online therapy.mp.

40 Digital therapy.mp.

41 Mobile Applications/

42 apps.mp.

43 smart pill dispensers.mp.

44 Text Messaging/

45 Web-based platforms.mp.

46 Wearable Electronic Devices/

47 Virtual support groups.mp.

48 online communities.mp.

49 31 or 32 or 33 or 34 or 35 or 36 or 37 or 38 or 39 or 40 or 41 or 42 or 43 or 44 or 45 or 46 or 47 or 48

50 10 and 18 and 30 and 49

**CINAHL Plus**

(neoplasms or tumor or cancer or carcinoma or oncology or oncolytic)

AND

(antineoplastic agents or anti-cancer treatment or anti-cancer therapies or medication or tablet or capsule or oral drug or pill or oncolytic agent)

AND

(adherence or compliance or non-adherence or noncompliance or treatment adherence or treatment compliance or medication adherence or patient compliance or treatment refusal or self administration or non-adherence or non-compliance or persistence or concordance)

AND

(telemedicine or telehealth or ehealth or e-health or mhealth or m-health or digital or digital interventions or digital health or internet-based interventions or online therapy or digital therapy or mobile applications or apps or smart pill dispensers or text messaging or web-based platforms or wearable electronic devices or virtual support groups or online communities or automated intervention)
