# Supplementary material for: The Efficacy of Digital Interventions on Adherence to Oral Systemic Anticancer Therapy Among Patients With Cancer: Systematic Review and Meta-Analysis
Source: JMIR Cancer. 2025 Apr 16;11:e64208. doi: 10.2196/64208 (PMC12017607; doi:10.2196/64208)
Supplement: Multimedia Appendix 3 [file cancer-v11-e64208-s003.pdf]

Appendix 3. The risk-of-bias assessment for the randomized controlled trials

Author, year, country	Bias arising from the randomization process	Bias due to deviations from intended intervention	Bias due to missing outcome data	Bias in the measurement of the outcome	Bias in the selection of the reported result	Overall
Kekale, 2016, Finland	Some concerns	Low risk	Some concerns	Some concerns	Low risk	Some concerns
Kim, 2018, Korea	Low risk	Low risk	Low risk	Some concerns	Low risk	Some concerns
Sikorskii, 2018, US	Low risk	Low risk	Some concerns	Low risk	Low risk	Some concerns
Eldeib, 2019, Egypt	Some concerns	Low risk	Low risk	Low risk	Low risk	Some concerns
Greer, 2020, US	Low risk	Low risk	Low risk	Some concerns	Low risk	Some concerns
Hershman, 2020, US	Low risk	Low risk	Some concerns	Some concerns	Low risk	Some concerns
Tan, 2020, Singapore	Low risk	Low risk	Low risk	Some concerns	Low risk	Some concerns
Bouleftour, 2021, France	Low risk	Low risk	Low risk	Some concerns	Low risk	Some concerns
Karaaslan-Eser, 2021, Turkey	Low risk	Low risk	Some concerns	Some concerns	Low risk	Some concerns
Mir, 2022, France	Low risk	Low risk	Some concerns	Some concerns	Low risk	Some concerns
Park, 2022, Korea	Low risk	Low risk	Some concerns	Some concerns	Low risk	Some concerns
Singleton, 2023, Australia	Low risk	Low risk	Low risk	Some concerns	Low risk	Some concerns
Guio, 2024, US	Low risk	Low risk	Some concerns	Some concerns	Low risk	Some concerns

[a]The risk-of-bias tool for randomized trials (RoB 2) was applied. Background color caption: green: low risk of bias; yellow: some concerns; and red: high risk of bias.
