# Supplementary material for: The Efficacy of Digital Interventions on Adherence to Oral Systemic Anticancer Therapy Among Patients With Cancer: Systematic Review and Meta-Analysis
Source: JMIR Cancer. 2025 Apr 16;11:e64208. doi: 10.2196/64208 (PMC12017607; doi:10.2196/64208)
Supplement: Multimedia Appendix 4 [file cancer-v11-e64208-s004.docx]

Appendix 4. Characteristics of interventions

| **Author, year, country** | **Intervention component** | **Key contents** | **Delivery** |
| --- | --- | --- | --- |
| Kekale, 2016, Finland [46] | Nurse-led medication counselling, an information booklet, video and website, and text message reminders. | - Understanding chronic myeloid leukemia as a disease. - Establishing treatment goals for tyrosine kinase inhibitors therapy. - Emphasizing the significance of adhering to tyrosine kinase inhibitors medication as directed. - Empowering patients in self-managing adverse drug reactions. | Trained hematology nurses provided counselling |
| Kim, 2018, South Korea [22] | Mobile game (ILOVEBREAST) developed by the CLGAMES | Educational content on   - Preventing side effects of anti-cancer drugs. - Assistance in managing side effects. - Promotion of a positive mood. - Engagement in activities with the hope to encourage participation in similar real-life activities. | Patient self-administered |
| Sikorskii, 2018, US^a^ [33] | Phone call to remind medication taking and symptom management | - Reminders to take their medication. - Evaluate 18 symptoms. - Directed patients to a Medication Management and Symptom Management Toolkit. | Interactive voice response system |
| Eldeib, 2019, Egypt [23] | Telephone-based follow-up on top of the standard care | - Documenting, assessing and grading adverse effects. - Providing appropriate strategies. - Directly querying patients about their adherence. - Reinforcing the significance of adhering to the prescribed oral capecitabine regimen. | Principal investigator |
| Greer, 2020, US^a^ [43] | Smartphone application (with the ability to integrate wearable physical activity tracking) | - Customized medication dosing timetables. - A module for adherence and symptom reporting. - Educational materials covering symptom management. - Information for various cancer-related subjects. - Fitbit integration for monitoring physical activity. | Patient self-administered |
| Hershman, 2020, US^a^ [21] | Educational text messages | - Addressing obstacles to medication adherence. - Prompts for action. - Statements concerning medication efficacy. - Reinforcements for patients to take their medications. | CareSpeak  Communications |
| Tan, 2020, Singapore [48] | SMS^b^ reminders | The message read:  <PATIENT NAME>, please be reminded to take your anti-cancer medicine as instructed by your doctor. Take one tablet once every day. | Provided by a company (unspecified) |
| Bouleftour, 2021, France [44] | Nurse-led telephone-based follow-up | - Identify potential toxicities. - Management strategies and support for self-management of potential toxicities. - Directly querying patients about their adherence. | Four trained nurses |
| Karaaslan-Eser, 2021, Turkey [25] | Smartphone application (OKTED) | - Record symptoms and severity. - Recommendations and information for the management of relevant symptoms. - Provide medication reminders. | Researcher-installed and patient self-administered |
| Mir, 2022, France [45] | Nurse navigator-led follow-up system integrated with the CAPRI^c^ web portal and smartphone application | - Record and track data. - Contact nurse navigators via a secure messaging system. - Review treatment and side-effect information. - Report symptoms. - Healthcare professionals could log on to the web portal, view the patients' information, and communicate with nurse navigators. | Two nurse navigators |
| Park, 2022, South Korea [24] | A smart pill bottle reminder syncing with the Pillsy mobile application (Pillsy, Inc., Seattle, WA) via Bluetooth | The smart pill bottle was equipped with blinking reminders to alert users of the time to take medication.  The mobile application served numerous functions:   - Recording the users' dose when the bottle cap is opened. - Tracking remaining medications. - Alarm reminders. - Sending notifications to designated helpers when users miss a dose. | Patient self-administered |
| Singleton, 2023, Australia [47] | Lifestyle-focused text message intervention | - Physical activity. - Maintaining a healthy diet. - Promoting social and emotional wellbeing. - Guidance on medication adherence. - Managing side effects. - Offering general information related to breast cancer. | EMPOWER-SMS^b^ |
| Guio, 2024, Colombia [14] | Digital education is delivered by an interactive virtual platform with personalized multimedia (video, text, audio, image) displayed as short slideshows | The topics covered include:   - Pathophysiology, diagnosis, follow-up, prognosis tests, therapeutic options and the importance of treatment adherence, side effects of treatment, and use of electronic resources. - Pain management. - Use of alternative and complementary medicine. - Performing daily activities. - Recommendations on lifestyle changes, and nutrition. - Sexual activity. - Importance of adherence to maintenance treatment. - Strategies to prevent relapses and complications. - Decision-making at the end of life and the role of caregivers. | Unspecified |

^a^US: United States. ^b^SMS: short message service. ^c^CAPRI: Impact of a Monitoring Device for Patients with Cancer Treated Using Oral Therapeutics.
